# Successful Surgical Treatment of Hemosuccus Pancreaticus Caused by Rupture of a Transverse Pancreatic Artery Aneurysm: A Case Report

**DOI:** 10.70352/scrj.cr.25-0538

**Published:** 2026-01-08

**Authors:** Ayaka Ogura, Fuminori Mihara, Mai Nakamura, Takashi Kokudo, Yuichiro Mihara, Fuyuki Inagaki, Takeyuki Watadani, Hideki Miyazaki, Toru Igari, Norihiro Kokudo

**Affiliations:** 1 Department of Surgery, Hepato-Biliary Pancreatic Surgery Division, National Center for Global Health and Medicine, Japan Institute for Health Security, Tokyo, Japan; 2 Department of Radiology, National Center for Global Health and Medicine, Japan Institute for Health Security, Tokyo, Japan; 3 Department of Pathology, National Center for Global Health and Medicine, Japan Institute for Health Security, Tokyo, Japan

**Keywords:** aneurysm, pancreatitis, gastrointestinal hemorrhage, pancreatectomy, angiography, transverse pancreatic artery, hemosuccus pancreaticus

## Abstract

**INTRODUCTION:**

A pancreatic pseudoaneurysm is a rare but potentially life-threatening complication of pancreatitis. Although pseudoaneurysms typically arise from the splenic, gastroduodenal, or pancreaticoduodenal arteries, transverse pancreatic artery involvement is uncommon. Here, we report the case of a pseudoaneurysm in the transverse pancreatic artery that presented with repeated episodes of obscure gastrointestinal bleeding over an extended period, with a clinical course suggestive of pancreatic duct rupture.

**CASE PRESENTATION:**

A 49-year-old male with chronic alcohol-related pancreatitis was brought to our hospital via ambulance because of abdominal pain and lower gastrointestinal bleeding. He had a history of recurrent obscure gastrointestinal bleeding for >11 years, with no source identified despite repeated upper and lower endoscopies, capsule endoscopy, and double-balloon enteroscopy. On admission, the patient was hemodynamically stable and had mild anemia. Contrast-enhanced CT revealed pancreatic calcifications, and upper endoscopy revealed bleeding from the major duodenal papilla. Angiography revealed a pseudoaneurysm in a tortuous branch of the transverse pancreatic artery. Coil embolization was attempted but could not be completed due to anatomical complexity. Rebleeding occurred during the procedure, prompting an emergency distal pancreatectomy and splenectomy. Surgical resection was achieved, and the patient recovered uneventfully with no recurrent bleeding at 6 months of follow-up.

**CONCLUSIONS:**

Although rare, pseudoaneurysms arising from the transverse pancreatic artery can cause life-threatening hemorrhages in the pancreatic duct. In such cases, early recognition, prompt angiographic investigation, and appropriate surgical intervention are critical for successful management.

## INTRODUCTION

Pancreatic pseudoaneurysms are serious complications of pancreatitis, and their rupture can lead to life-threatening hemorrhage.^1)^ Generally, diagnosis involves contrast-enhanced CT and angiography, and treatment options include endovascular embolization or surgical resection.^2)^ The most common sites of pseudoaneurysm formation are the splenic, gastroduodenal, and pancreaticoduodenal arteries.^3)^ Previous studies have suggested that management strategies should be tailored according to the type of artery involved.^4)^ However, reports of pseudoaneurysms arising from the transverse pancreatic artery are limited, and optimal management strategies remain unclear.

Here, we report the case of a ruptured pseudoaneurysm of the transverse pancreatic artery that resulted in hemosuccus pancreaticus. The patient presented with recurrent obscure gastrointestinal bleeding. Hemosuccus pancreaticus is defined as bleeding into the pancreatic duct, which drains into the duodenum via the ampulla of Vater, often leading to gastrointestinal bleeding that can be difficult to localize.^5)^ Hemosuccus pancreaticus is rarely a cause of gastrointestinal bleeding with an estimated incidence of 0.07% and a reported mortality rate up to 10%, rising to as high as 90% when treated conservatively.^5)^ These data underscore the importance of early diagnosis and intervention. This case highlights the importance of prompt angiographic evaluation and emphasizes the need for close collaboration between interventional radiologists and surgeons, with a readiness for surgical intervention when endovascular treatment is challenging.

## CASE PRESENTATION

A 49-year-old male with a history of alcohol-related chronic pancreatitis was brought to our hospital via emergency medical services. He presented with abdominal pain and lower gastrointestinal bleeding. His alcohol consumption is approximately 200 g per day for several years.

During the previous 11 years, the patient experienced 3 episodes of lower gastrointestinal bleeding, each following an acute exacerbation of chronic pancreatitis. Conservative management for pancreatitis at other hospitals led to clinical improvement. However, despite multiple assessments using contrast-enhanced CT, upper endoscopy, colonoscopy, small bowel capsule endoscopy, and double-balloon enteroscopy, the source of bleeding remained unidentified. Each episode of bleeding resolved spontaneously.

On arrival at the emergency department, the patient was hemodynamically stable. Digital rectal examination revealed bright red blood, and laboratory testing showed a hemoglobin level of 112 g/L, consistent with mild anemia.

**Fig. 1:**
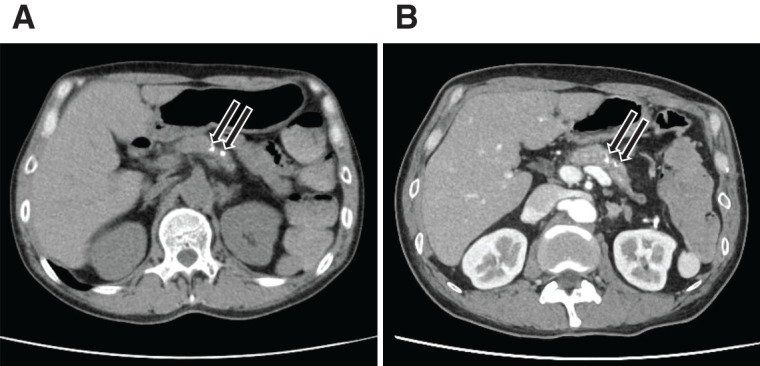
Preoperative CT imaging. (**A**) Unenhanced CT reveals hyperdense areas within the pancreas suggestive of calcifications (arrows). (**B**) Contrast-enhanced CT shows the same hyperdense areas at the corresponding site (arrows), which is also suggestive of pancreatic calculi, with no evidence of active extravasation.

Unenhanced multidetector CT and dynamic contrast-enhanced multidetector CT revealed pancreatic calcifications consistent with chronic pancreatitis (**Fig. 1A** and **1B**). Active contrast extravasation or vascular abnormalities were not observed.

**Fig. 2:**
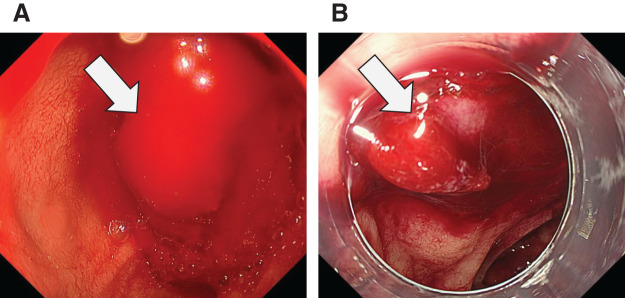
Upper gastrointestinal endoscopy. (**A**) Upon insertion of the endoscope, a large amount of blood was noted near the major duodenal papilla (arrow). (**B**) Following irrigation, active hemorrhage was identified originating from the major duodenal papilla (arrow).

Subsequent upper gastrointestinal endoscopy revealed active bleeding from the major duodenal papilla, raising a suspicion of pancreatic duct hemorrhage (**Fig. 2A** and **2B**).

**Fig. 3:**
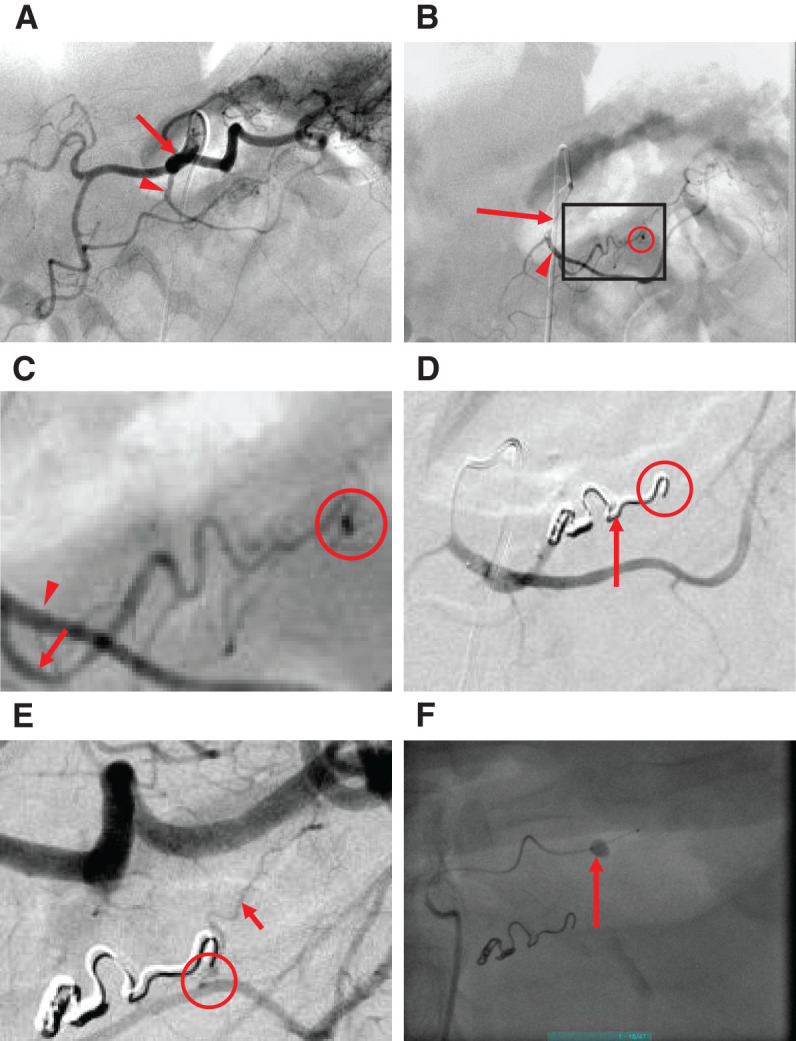
Angiographic findings. (**A**) Selective angiography demonstrated the dorsal pancreatic artery (arrowhead) arising from the celiac artery (arrow). (**B**) Selective angiography demonstrated the transverse pancreatic artery (arrowhead) branches from the dorsal pancreatic artery (arrow), and a distal branch of the transverse pancreatic artery gave rise to a small aneurysm (circled). (**C**) Magnified view of the area outlined in (**A**), showing a branch arising from the transverse pancreatic artery (arrowhead) with a small aneurysm (circled) at its distal end (arrow). (**D**) Coil embolization (arrow) of the aneurysm arising from the branch of the transverse pancreatic artery. Post-embolization angiography via the dorsal pancreatic artery showed no residual aneurysm filling. (**E**) Angiography via the splenic artery demonstrates the residual aneurysm (circled) arising from the great pancreatic artery (arrow). (**F**) A balloon catheter (arrow) was emergently placed in the splenic artery.

Angiography clearly demonstrated the arterial anatomy surrounding the pancreas and confirmed the presence of a pseudoaneurysm. Selective angiography of the dorsal pancreatic artery, which originated from the celiac trunk (**Fig. 3A**), revealed a pseudoaneurysm approximately 1.5 mm in diameter arising from a small, tortuous branch of the transverse pancreatic artery. The transverse pancreatic artery itself originated from the dorsal pancreatic artery (**Fig. 3B** and **3C**). At the start of the angiographic procedure, the patient’s blood pressure was 143/87 mmHg and heart rate was 76 beats/min. These findings were suggestive of hemosuccus pancreaticus. This branch was extremely narrow and tortuous, and although coil embolization was successfully delivered into the aneurysm, anatomical limitations prevented access to the distal segment (**Fig. 3D**). Therefore, angiography via the splenic artery was performed, which demonstrated retrograde filling of the pseudoaneurysm through the great pancreatic artery with collateral communication to the previously embolized branch of the transverse pancreatic artery (**Fig. 3E**). While attempting additional coiling via this collateral pathway, the patient suddenly developed hematochezia and abdominal pain, suggesting a re-rupture of the pseudoaneurysm. The patient’s blood pressure increased to 199/105 mmHg, with a heart rate of 62 beats/min. Within 10 minutes, the patient’s vital signs partially stabilized, with a blood pressure of 146/98 mmHg and a heart rate of 84 beats/min. Although the patient did not progress to shock during this period, the risk of re-rupture and hemodynamic collapse remained a concern. To achieve hemostasis and stabilize the patient, temporary balloon occlusion of the splenic artery was performed to reduce blood flow into the pseudoaneurysm (**Fig. 3F**).

**Fig. 4:**
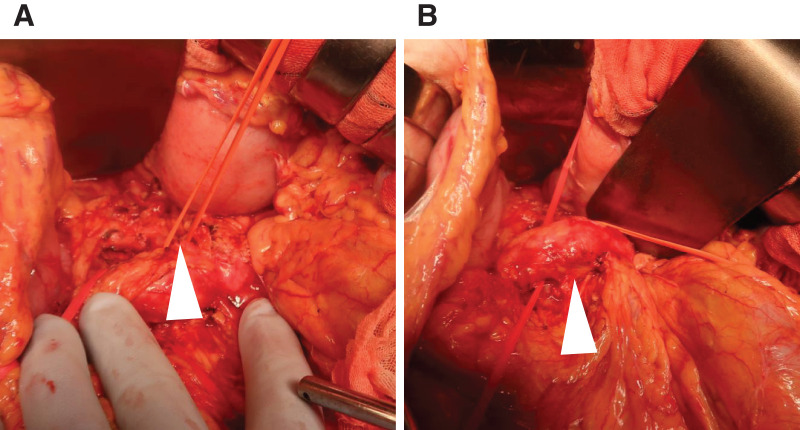
Intraoperative findings. (**A**) Taping of the splenic artery (arrowhead). (**B**) Tunneling of the pancreas (arrowhead).

Given the risk of continued bleeding and anatomical complexity precluding complete embolization, the patient was immediately transferred to an operating room. Emergency distal pancreatectomy and splenectomy were performed. The splenic artery was dissected and looped with vessel tape intraoperatively (**Fig. 4A**). After deflation and removal of the balloon catheter under direct visualization, the artery was ligated and divided.

The pancreas was then taped and transected just above the superior mesenteric vein, which is the standard transection line for distal pancreatectomy (**Fig. 4B**). Based on the angiographic findings, the aneurysm was assumed to be located distal to this line, and the resection was planned so that the aneurysm would be included in the resected specimen.

Dense fibrosis of the pancreatic parenchyma related to chronic pancreatitis resulted in moderate bleeding during dissection. Notably, intraoperative bleeding was well controlled, and no excessive hemorrhage occurred. The operation time was 169 minutes, and the intraoperative blood loss was 670 mL.

The patient recovered uneventfully and was discharged in good general condition on POD 13. No episodes of recurrent gastrointestinal bleeding were observed during the 6-month follow-up. Following surgery, the patient successfully abstained from alcohol consumption.

## DISCUSSION

This case report has 2 important clinical implications. First, early surgical intervention may be required when a pancreatic pseudoaneurysm arises from the transverse pancreatic artery. Second, angiographic evaluations should be performed in patients with a history of pancreatitis who present with recurrent obscure gastrointestinal bleeding. This case was characterized by a small pseudoaneurysm arising from an uncommon location and intermittent gastrointestinal bleeding due to hemorrhage into the pancreatic duct. These factors contributed to difficulty identifying the bleeding source over an extended period. To the best of our knowledge, this is the first reported case in which a pseudoaneurysm arising from the transverse pancreatic artery led to hemosuccus pancreaticus and presented as recurrent gastrointestinal bleeding. Although 4 cases of pancreatitis-associated pseudoaneurysms of the transverse pancreatic artery have been previously reported, none have described a clinical course consistent with hemosuccus pancreaticus^6–9)^ (**Table 1**).

**Table 1:** Previously reported cases of pseudoaneurysm arising from the transverse pancreatic artery

Reference	Year	Age/Sex	GI bleed	Stomach Pain	Detection of pseudoaneurysm on CT or CTA	Origin of TPA	Size of aneurysm	Angiographic treatment	Percutaneous treatment	Surgery	Postoperative complications
Takematsu et al.^6)^	2021	66/F	–	+	+	Splenic artery	5 mm	Attempted but failed	None	Emergency aneurysmectomy + ligation	None
Masatsugu et al.^7)^	2000	64/M	–	+	+	–	5 mm	Attempted but failed	None	DP + splenectomy	None
Knight et al.^8)^	1982	28/M	–	+	– Only pseudocyst	Celiac trunk	Not specified	Successful	None	None	None
Pacheco Jiménez et al.^9)^	2011	74/M	+ Gastric ulcer	–	+	Superior mesenteric artery	3 cm	Not feasible	Thrombin injection ×2	None	None

DP, distal pancreatectomy; GI, gastrointestinal; TPA, transverse pancreatic

First, when a pancreatic pseudoaneurysm arises from the transverse pancreatic artery, early surgical intervention may be required because of the anatomical complexity and risk of rebleeding. Endovascular therapy is generally considered the first-line treatment for pancreatic pseudoaneurysms, and surgical treatment is reserved for cases in which endovascular intervention is anatomically or hemodynamically difficult.^2,3)^ The reported success rates of endovascular interventions is 75%–100%. However, pseudoaneurysms originating from the transverse pancreatic artery present a significantly greater challenge for treatment via endovascular methods. Among the 4 previously documented cases of pseudoaneurysm and the present case, endovascular intervention was successful in only 2 of the 5 instances (40%), with the remaining 3 cases requiring surgical intervention (**Table 1**).

In this case, 2 factors necessitated surgical intervention. First was the anatomical complexity of the transverse pancreatic artery. The pseudoaneurysm originated from a thin and tortuous branch of this artery. Moreover, the artery receives blood from both the dorsal and great pancreatic arteries, which are branches of the splenic artery. Although the initial coil embolization was achieved via the dorsal pancreatic artery, blood flow to the aneurysm persisted, requiring additional embolization through the great pancreatic artery. Previous anatomical studies have shown that the transverse pancreatic artery typically arises from multiple sources (most often the gastroduodenal artery) and runs along the inferior surface of the pancreatic body and tail.^10)^ Furthermore, the transverse pancreatic artery frequently forms anastomoses with the dorsal and great pancreatic arteries and may create an arterial arcade.^10,11)^ This complexity of anatomy likely contributed to the technical challenges encountered during endovascular treatment in this case. Second was the hemodynamic instability caused by hemorrhage into the pancreatic duct. It is well known that pseudoaneurysms exposed to pancreatic juice become increasingly fragile due to enzymatic arterial wall digestion, which increases the risk of rupture.^5)^ In this case, upper gastrointestinal endoscopy revealed bleeding from the duodenal papilla, indicating hemorrhage into the pancreatic duct. Additionally, the patient developed acute abdominal pain during endovascular treatment, strongly suggesting a re-rupture of the pseudoaneurysm. Based on these considerations, surgeons should anticipate a risk of failure in endovascular treatment for pseudoaneurysms originating from the transverse pancreatic artery and should be prepared to promptly proceed with surgical conversion when deemed necessary.

In patients with a history of pancreatitis who present with recurrent obscure gastrointestinal bleeding, angiographic evaluation should be performed at an early stage, even when conventional imaging studies are negative. The differential diagnoses of obscure gastrointestinal bleeding include angiodysplasia, small intestinal tumors, Meckel’s diverticulum, Crohn’s disease, enteropathy caused by non-steroidal anti-inflammatory drugs, bleeding from the biliary tract, aorto-enteric fistula, and hemorrhage into the pancreatic duct.^12)^ Among these, hemorrhage into the pancreatic duct can cause life-threatening bleeding due to rupture of a pancreatic pseudoaneurysm, which requires particular attention.^5)^ Various diagnostic modalities are used to distinguish these conditions, including contrast-enhanced CT, capsule endoscopy, double-balloon enteroscopy, CT or magnetic resonance enterography, angiography, endoscopic retrograde cholangiopancreatography, endoscopic ultrasonography, and CT angiography.^13–19)^ For detecting hemorrhage into the pancreatic duct, angiography is considered the most sensitive diagnostic method, whereas upper and lower endoscopy fail to identify the bleeding source in more than 50% of cases.^5,20)^ In the present case, contrast-enhanced CT revealed only pancreatic calcifications, without evidence of contrast extravasation or a pseudoaneurysm. Additionally, multiple upper, lower, and small intestinal endoscopic examinations were performed before transfer to our institution; however, no bleeding source was successfully identified. It was only during the emergency admission that upper gastrointestinal endoscopy revealed active bleeding from the major duodenal papilla, prompting angiographic evaluation and leading to a definitive diagnosis. The pseudoaneurysm identified on angiography measured 1.5 mm in diameter, and the originating branch of the transverse pancreatic artery measured only 0.5 mm in diameter. Its detection on contrast-enhanced CT was likely missed because of the very small sizes of both the lesion and its feeding vessels, as previous reports have indicated that transverse pancreatic artery aneurysms of 5 mm or larger are generally detectable on CT^6,9)^ (**Table 1**).

This case highlights the importance of early recognition and multidisciplinary collaboration for the diagnosis and management of peripancreatic pseudoaneurysms. Furthermore, when pseudoaneurysms are associated with hemosuccus pancreaticus, these lesions are prone to hemodynamic instability. In such cases, early surgical intervention is essential to prevent fatal hemorrhagic shock. Additionally, in patients with a history of pancreatic disease who present with intermittent obscure gastrointestinal disease, the possibility of hemosuccus pancreaticus should always be considered, and early angiographic diagnosis is of critical importance.

## CONCLUSIONS

We encountered the case of a pseudoaneurysm arising from the transverse pancreatic artery that presented as hemosuccus pancreaticus. This case illustrates 2 important clinical lessons: pseudoaneurysms may require early surgical intervention owing to their anatomical complexity, and in patients with pancreatitis and recurrent obscure gastrointestinal bleeding, early angiographic evaluation should be performed, even when conventional imaging is inconclusive.

## DECLARATIONS

### Availability of data and materials

The datasets supporting the conclusions of this study are included in this article.

### Use of artificial intelligence tools

No, artificial intelligence tools were not used in the preparation of this manuscript.

## References

[1] Mitrovic Jovanovic M, Tadic B, Jankovic A, et al. Endovascular treatment of a pseudoaneurysm of the posterior inferior pancreaticoduodenal artery as a complication of chronic pancreatitis: a case report. J Int Med Res 2022; 50: 3000605221083441. 35225703 10.1177/03000605221083441 PMC8987367

[2] Sanchez Cruz C, Abera Woldehana N, Ponce-Lujan L, et al. Comprehensive review of surgical and radiological management of hemorrhagic pancreatitis: current strategies and outcomes. Cureus 2024; 16: e65064. 39171005 10.7759/cureus.65064 PMC11336159

[3] Evans RP, Mourad MM, Pall G, et al. Pancreatitis: preventing catastrophic haemorrhage. World J Gastroenterol 2017; 23: 5460–8. 28852306 10.3748/wjg.v23.i30.5460 PMC5558110

[4] Pang TC, Maher R, Gananadha S, et al. Peripancreatic pseudoaneurysms: a management-based classification system. Surg Endosc 2014; 28: 2027–38. 24519028 10.1007/s00464-014-3434-9 PMC4065337

[5] Tarar ZI, Khan HA, Inayat F, et al. Hemosuccus pancreaticus: a comprehensive review of presentation patterns, diagnostic approaches, therapeutic strategies, and clinical outcomes. J Investig Med High Impact Case Rep 2022; 10: 23247096211070388. 10.1177/23247096211070388 PMC8796068 35045737

[6] Takematsu T, Kosumi K, Tajiri T, et al. Surgical resection of a ruptured transverse pancreatic artery aneurysm. Surg Case Rep 2021; 7: 53. 33616793 10.1186/s40792-021-01128-4 PMC7900318

[7] Masatsugu T, Yamaguchi K, Yokohata K, et al. Hemorrhagic pseudocyst and pseudocyst with pseudoaneurysm successfully treated by pancreatectomy: report of three cases. J Hepatobiliary Pancreat Surg 2000; 7: 432–7. 11180866 10.1007/s005340070040

[8] Knight RW, Kadir S, White RI Jr. Embolization of bleeding transverse pancreatic artery aneurysms. Cardiovasc Intervent Radiol 1982; 5: 37–9. 7083260 10.1007/BF02552102

[9] Pacheco Jiménez M, Moreno Sánchez T, Moreno Rodríguez F, et al. Pancreatic tail pseudoaneurysm: percutaneous treatment by thrombin injection (in Spanish). Radiologia 2014; 56: 167–70. 21944714 10.1016/j.rx.2011.04.009

[10] Kimura W, Hirai I, Yamaguchi H, et al. Surgical anatomy of arteries running transversely in the pancreas, with special reference to the superior transverse pancreatic artery. Hepatogastroenterology 2004; 51: 973–9. 15239227

[11] Covantev S, Mazuruc N, Belic O. The arterial supply of the distal part of the pancreas. Surg Res Pract 2019; 2019: 5804047. 31016226 10.1155/2019/5804047 PMC6446113

[12] Filippone A, Cianci R, Milano A, et al. Obscure and occult gastrointestinal bleeding: comparison of different imaging modalities. Abdom Imaging 2012; 37: 41–52. 21912990 10.1007/s00261-011-9802-1

[13] Sakai E, Ohata K, Nakajima A, et al. Diagnosis and therapeutic strategies for small bowel vascular lesions. World J Gastroenterol 2019; 25: 2720–33. 31235995 10.3748/wjg.v25.i22.2720 PMC6580356

[14] Vlachou E, Koffas A, Toumpanakis C, et al. Updates in the diagnosis and management of small-bowel tumors. Best Pract Res Clin Gastroenterol 2023; 64-65: 101860. 37652650 10.1016/j.bpg.2023.101860

[15] Kuru S, Kismet K. Meckel’s diverticulum: clinical features, diagnosis and management. Rev Esp Enferm Dig 2018; 110: 726–32. 30032625 10.17235/reed.2018.5628/2018

[16] Veauthier B, Hornecker JR. Crohn’s disease: diagnosis and management. Am Fam Physician 2018; 98: 661–9. 30485038

[17] Shin SJ, Noh CK, Lim SG, et al. Non-steroidal anti-inflammatory drug-induced enteropathy. Intest Res 2017; 15: 446–55. 29142512 10.5217/ir.2017.15.4.446 PMC5683975

[18] Merrell SW, Schneider PD. Hemobilia--evolution of current diagnosis and treatment. West J Med 1991; 155: 621–5. 1812632 PMC1003111

[19] Chung J. Management of aortoenteric fistula. Adv Surg 2018; 52: 155–77. 30098611 10.1016/j.yasu.2018.03.007

[20] Shetty S, Shenoy S, Costello R, et al. Hemosuccus pancreaticus. J Ayub Med Coll Abbottabad 2019; 31: 622–6. 31933323

